# Nickel–Molybdenum-Based Three-Dimensional Nanoarrays for Oxygen Evolution Reaction in Water Splitting

**DOI:** 10.3390/molecules29163966

**Published:** 2024-08-22

**Authors:** Zhi Lu, Shilin Li, Yuxin Wang, Jiefeng Wang, Yifan Guo, Jiaqi Ding, Kun Tang, Yingzi Ren, Long You, Hongbo Meng, Guangxin Wang

**Affiliations:** 1 School of Materials Science and Engineering, Henan University of Science and Technology, Luoyang 471003, China; lishilin0402@163.com (S.L.); wangyx202403@163.com (Y.W.); guoyifan20010310@163.com (Y.G.); d2827868483@163.com (J.D.); tang01101344@163.com (K.T.); m18177395505@163.com (Y.R.); yonglong0@163.com (L.Y.); 2 Henan Engineering Research Center for High Purity Materials and Sputtering Targets, Luoyang 471003, China; 3 School of Mechanical Engineering, Anyang Institute of Technology, Anyang 455099, China; wangyufan2007@163.com; 4 Luoyang Crystal Union Photoelectric Materials Co., Ltd., Luoyang 471100, China; hongbomeng@163.com

**Keywords:** nickel–molybdenum, OER, electrocatalyst, water splitting

## Abstract

Water splitting is an important approach to hydrogen production. But the efficiency of the process is always controlled by the oxygen evolution reaction process. In this study, a three-dimensional nickel–molybdenum binary nanoarray microstructure electrocatalyst is successfully synthesized. It is grown uniformly on Ni foam using a hydrothermal method. Attributed to their unique nanostructure and controllable nature, the Ni-Mo-based nanoarray samples show superior reactivity and durability in oxygen evolution reactions. The series of Ni-Mo-based electrocatalysts presents a competitive overpotential of 296 mV at 10 mA·cm^−2^ for an OER in 1.0 M KOH, corresponding with a low Tafel slope of 121 mV dec^−1^. The three-dimensional nanostructure has a large double-layer capacitance and plenty of channels for ion transfer, which demonstrates more active sites and improved charge transmission. This study provides a valuable reference for the development of non-precious catalysts for water splitting.

## 1. Introduction

Serious environmental pollution and energy crises have resulted from the excessive consumption of fossil fuels. Renewable energy has become urgent for the sustainable development of society [1,2]. As a clean energy source, hydrogen can be converted from other green energy such as wind and solar, and is convenient to transport by pipeline or a compressed air accumulator. Thus, it is considered an ideal substitute to take the place of fossil fuels [3,4]. Compared with other methods, using sustainable sources, water splitting is a sustainable route of hydrogen generation. Its efficiency is mainly determined by the OER process which occurs in the anode [5]. Thus, determining how to considerably improve the efficiency of the OER in the anode has become critical for reducing the cost of water splitting. Catalysts are one of the optimal choices [6,7]. At present, several types of catalysts including noble metals (for example, Ru and Pt) and their oxides (for example, RuO_2_) were designed for OERs and have been proven to be effective and stable in application. Their low reserves lead to their high cost, which limits their industrial application severely [8,9].

It is necessary to search for high-efficiency and stable low-cost candidates to replace noble metals. So far, some earth-abundant metal hydroxides have attracted much interest from researchers due to their excellent performance as electrocatalysts in water splitting [10,11]. For example, Ni-based LDHs and Co-based LDHs show superior conductivity and electronic properties in OERs [12,13]. Because monometallic hydroxides cannot easily achieve the expected performance for OERs due to their inherent deficiency [14], bimetallic hydroxides may adjust the electronic structure of the electrode surface and contribute to a significant improvement in OER performance [15]. Wei et al. constructed a Ni-Ir-based catalyst using electrochemical deposition technology. The Ir atoms were anchored precisely on the three-fold facial center cubic hollow sites of the Ni hydroxides, which contributed to providing more covalent bonds between oxygen atoms and Ir atoms. This led to a low OER overpotential of about 228 mV under 10 mAcm^−2^ [16]. Gao et al. constructed a 2D/1D/NF-structure electrocatalyst; the 2D structure was composed of NiV-based nanoflakes which intercalated with B(OH)^4−^, and the 1D structure was composed of NiCoP nanowires [17]. The heterostructures could provide sufficient diffusion channels for the electrolytes and gasses, and the mass transport and electron transfer in the reaction were improved significantly. The optimized structure contributed to weakening the negative influence of some anions, such as Cl^−^, for the hydrolysis of seawater. Cai et al. synthesized a novel electrocatalyst of NiOOH/(LDH/α-FeOOH) using a simple hydrothermal and an electro-oxidation treatment [18]. There was obviously charge transfer between the amorphous NiOOH-LDH and α-FeOOH. The overpotential of this electrocatalyst at 10 mA cm^−2^ was as low as 195 mV for OERs. These works proved that the promotion of electrocatalysts could be achieved through the precise construction of some new phases in the microstructure. Chen et al. designed a RuO_2_/NiFe electrocatalyst using a low-cost hydrothermal method [19]. The interaction between Ru atoms and the transition metal atoms on the hydroxides changed the microelectronic structure of the electrocatalyst, and contributed to a low overpotential and considerable activity for water splitting. These studies confirmed that the dopant atoms changed the electronic structures of the prepared metal hydroxides. Doping can change not only the intrinsic properties of the composite, but also the morphology of the composite, which affect the performance of the electrocatalyst significantly [20,21]. Regulating the structure of the electrocatalysts is also an efficient path to improving catalytic activity. Many two-dimensional-structure LDHs have been designed; their special structures give them low density, a high specific surface area, more exposed active sites, high morphological anisotropy, a unique electronic structure and fast charge/mass transport. This characteristic will enhance their catalytic performance [22,23]. Umeshbabu et al. synthesized a hierarchical flower-like NiCo_2_O_4_ based on multiwalled carbon nanotubes without a surfactant. The electrocatalyst showed 209 mV and 350 mV for an HER and an OER at 10 mA cm^−2^ in 0.1 M KOH, and its conductivity was six times greater than that of pure NiCo_2_O_4_. The nanostructure morphology provided outstanding stability [24]. Liu et al. designed Co_3_O_4_ nanocube nanoparticles, and they found that the transformation of the Co_3_O_4_ nanoparticles’ surface into a highly active OER catalytic phase can be promoted by Pt support [25]. Tamboli et al. prepared a Mo-doped NiFe-LDH electrocatalyst. The results showed that the metal dopants changed the ΔG_OH_ and ΔG_O_-ΔG_OH_ at the Ni-sites, which can be rationalized by the difference in electron affinity between Ni^3+^ and Fe^3+^ sites. Then, the electronic structure and active site density of the catalyst were modulated by the metal dopants and led to enhanced catalytic activity and stability [26].

In this work, we studied Mo-doped nickel-based nanoarrays with a regulable morphology and composition. The hydrothermal process is low-cost and benefits the regulation of a microstructure, and even affects its performance in the following ways. The morphology and microstructure of nanomaterials can be adjusted by controlling the Mo/Ni ratio. Three-dimensional nanoarrays can provide more active sites, large double-layer capacitance and more channels for ion transfer, which benefit to the OER in the hydrolysis of water. The optimized NiMo-based nanoarrays exhibited competitive activity and catalytic stability. The overpotential of the electrocatalyst at 10 mAcm^−2^ was 296 mV with a Tafel slope of 121 mV dec^−1^ in 1.0 M KOH for the OER. It was mostly composed of transition metals, which contribute to reducing its cost and increasing its benefit to industrial application. This work can provide a reference for the design of novel Ni-based electrocatalysts.

## 2. Results and Discussion

### 2.1. Structure Characterization

**Figure 1:**
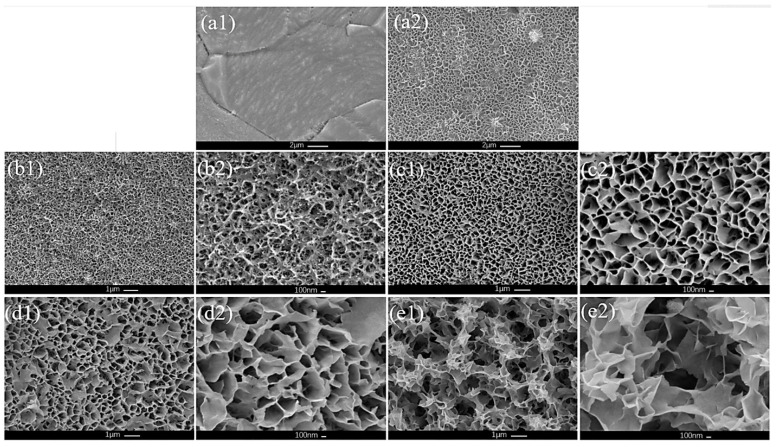
Morphology of (**a1**) pretreated Ni foam and (**a2**) NF surface after preparation. Low magnification of (**b1**) Ni-hydroxide/NF, (**c1**) Ni_2_Mo_1_-nanoarrays/NF, (**d1**) Ni_1_Mo_1_-nanoarrays/NF and (**e1**) Ni_2_Mo_3_-nanoarrays/NF. High magnification of (**b2**) Ni-hydroxide/NF, (**c2**) Ni_2_Mo_1_-nanoarrays/NF, (**d2**) Ni_1_Mo_1_-nanoarrays/NF and (**e2**) Ni_2_Mo_3_-nanoarrays/NF.

The Ni-Mo-based nanoarrays were prepared using a hydrothermal method. As shown in Figure 1(a1), the pretreated Ni foam had a smooth surface. While after the hydrothermal treatment, the Ni foam become obviously rough, the synthesized Ni-Mo-based nanoarrays showed a nanoporous structure (Figure 1(a2)).

**Figure 2:**
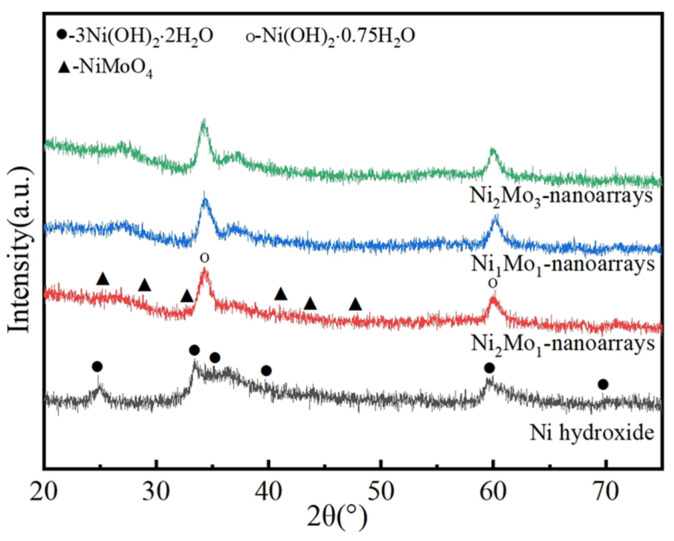
XRD patterns of NiMo-based nanoarrays.

The crystal structure of the Ni-Mo-based nanoarrays was identified through XRD. Figure 2 shows the XRD patterns of Ni-Mo-based nanoarrays with different Ni/Mo ratios. There are obvious differences in the patterns. When there is no Mo, the characteristic diffraction peaks in the Ni hydroxide center at 24.81°, 33.30°, 35.19°, 39.12°, 59.60° and 70.78° represent the lattice planes of (002), (110), (111), (200), (300) and (220) of 3Ni(OH)_2_·2H_2_O(JCPDNo.22-0444). With an increasing Mo/Ni ratio, the characteristic diffraction peaks in the NiMo-based nanoarray center at 25.33°, 28.82°, 32.58°, 41.23° and 47.41 represent the ($\bar{1}$12), (220), (022), (140) and ($\bar{2}$04) planes of NiMoO_4_ (JCPDS No.33-0948), while there are also (012) and (110) planes of Ni(OH)_2_·0.75H_2_O (JCPDS No.38-0715). This indicates the formation of Ni and Mo compounds in NiMo-based nanoarrays, which may bring changes in their microstructure. Given the above, according to the database of JCPDS, the main phase of Ni-hydroxide/NF is a hydroxide of hexagonal crystal structure. The main phase of NiMo-based nanoarrays involves the mixture of hydroxide and oxide, with the hydroxide being of a hexagonal crystal structure and the oxide of a monoclinic crystal structure.

From the overall SEM of NiMo-based nanoarrays (Figure 1(b1–e1)), with an increasing Mo/Ni ratio, the pore volume increases obviously. This contributes to an increase in the surface area for the catalytic reaction. Figure 1(b2–e2) show high magnification of the Ni-Mo-based electrocatalyst; as shown in these figures, the microstructure of the electrocatalyst changes significantly with increasing Mo. The microstructure develops from a fine mesh network into a porous nanosheet network, and the nanosheets and pores become even larger. This has significant influence on increasing the active sites, even for the OER performance. It is obvious that the microstructure of the Ni-Mo-based electrocatalyst can be regulated by adjusting the Mo/Ni ratio and obtaining special morphological features.

The proper tuning of the composition elements can regulate the surface characteristics and electronic structure of electrocatalysts [27]. This provides a reasonable strategy to enhance the catalytic behavior of catalysts [28]. The intrinsic electrical conductivity and the electron transfer process of binary metallic hydroxides are not excellent, but their unique nanoporous network and layered structures contribute to increasing exposed active sites and charge conductivity [29].

The pore volume and specific surface area of catalysts are affected by the ratio of specific ions, because many pores in the nanostructure are covered by newly formed nanocrystals [30]; thus, the morphology and catalytic activity of the materials are affected. Table 1 shows the average pore volume and specific surface area of the specimens. As shown in the table, Ni-hydroxide/NF, Ni_2_Mo_1_-nanoarrays/NF, Ni_1_Mo_1_-nanoarrays/NF and Ni_2_Mo_3_-nanoarrays/NF present average pore volumes of 0.016 cm^3^/g, 0.020 cm^3^/g, 0.019 cm^3^/g and 0.019 cm^3^/g. The average specific surface area of Ni-hydroxide/NF, Ni_2_Mo_1_-LDH/NF, Ni_1_Mo_1_-LDH/NF and Ni_2_Mo_3_-nanoarrays/NF are 11.08 m^2^/g, 14.63 m^2^/g, 13.30 m^2^/g and 13.07 m^2^/g, respectively. Therefore, an adequate increase in the Mo/Ni ratio contributes to improving the average pore volume and specific surface area of the NiMo-based nanoarrays and provides more catalytic active sites for catalysis.

The doping of Mo atoms may lead the energy band structure of the compounds to be modified, which helps to create a defect level in the band gap of the compounds of the electrocatalysts [31]. With the increase in dopant content, the energy gap of the compounds becomes narrowed, and because the defect level is close to the conduction band, the electrons in the defect level transfer into the conduction band of the compounds easily [31,32]. Then, the interaction between the dopant atoms and substrate atoms significantly improves the charge transport of electrocatalysts.

**Figure 3:**
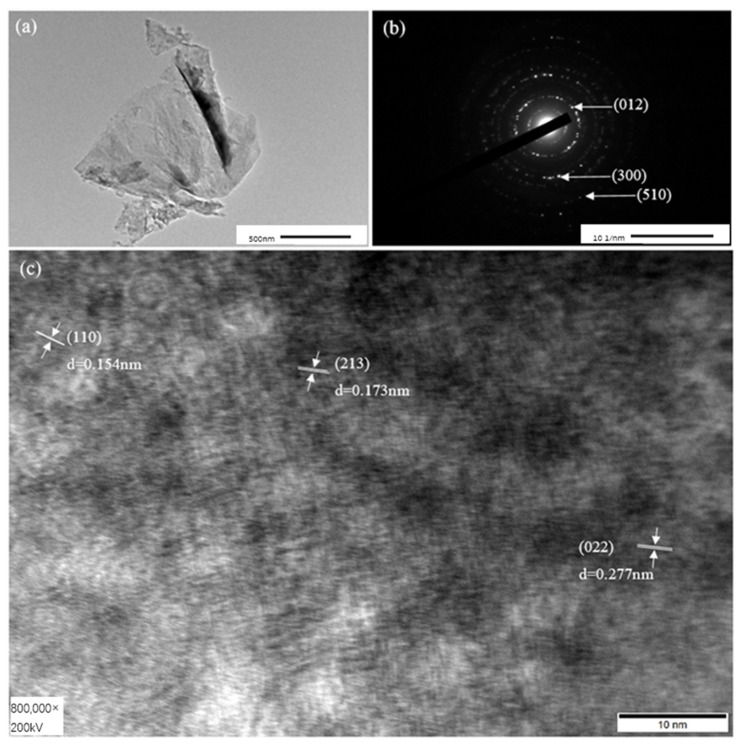
(**a**) TEM image, (**b**) SAED of Ni_2_Mo_1_-nanoarrays, and (**c**) high-resolution TEM image.

**Figure 4:**
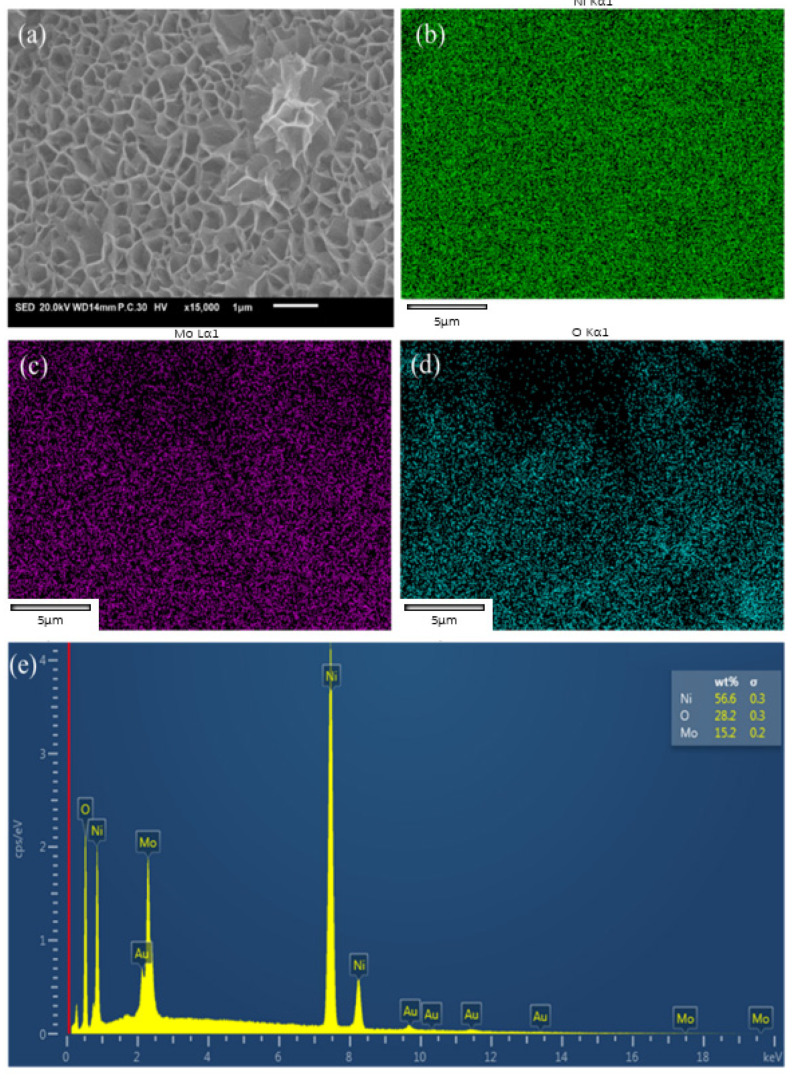
EDS of Ni_2_Mo_1_-nanoarray nanoporous network. (**a**) FESEM image of Ni_2_Mo_1_-nanoarray. EDS elemental mapping images of (**b**) Ni, (**c**) Mo and (**d**) O of Ni_2_Mo_1_-nanoarray. (**e**) EDS data of Ni_2_Mo_1_-nanoarray.

From the TEM image, the nanosheet of Ni_2_Mo_1_-nanoarrays has a considerable surface area (Figure 3a). This may benefit its catalytic behavior in water splitting. Figure 3c shows an HRTEM of Ni_2_Mo_1_-nanoarrays, in which the 0.154 nm interplanar distance corresponds to the (110) plane of Ni(OH)_2_·0.75H_2_O (JCPDS No.38-0715), and the 0.173 nm and 0.277 nm interplanar distances correspond to the (213) and (022) planes of NiMoO_4_ (JCPDS No.33-0948). Moreover, the characteristic diffraction spots detected in the selected area’s electron diffraction pattern correspond to the (012), (300) and (510) crystal planes of the microstructure (Figure 3b), which is consistent with the result of the XRD. EDS shows that the main elements are distributed uniformly on the nanosheets (Figure 4). This demonstrates the successful preparation of Ni-Mo-based nanoarrays with a nanoporous network.

For binary metal hydroxides, the intrinsic activity is difficult to improve; their surface area and active sites for OER can be increased through the regulation of their morphology [27]. Their porous structure can obviously increase the specific surface area of the electrocatalysts, which is conducive to improving the density of active sites of electrocatalysts. This is considered to affect many factors that are related to catalytic activity, including conductivity, thermal stability, absorption energy, mass transfer capacity, etc.; these are important to the OER of electrocatalysts.

**Figure 5:**
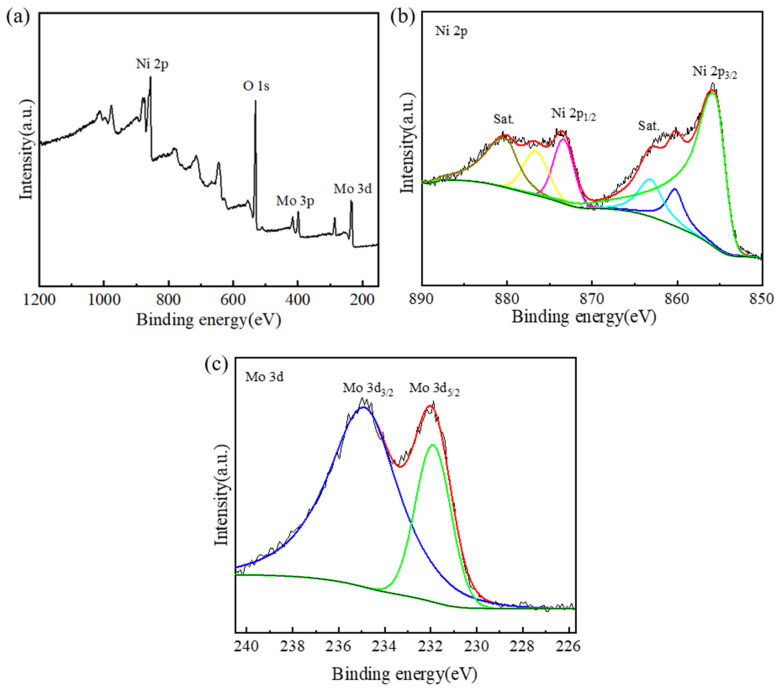
XPS spectra of Ni_2_Mo_1_-nanoarrays: (**a**) survey spectrum, (**b**) Ni 2p and (**c**) Mo 3d. The different colored lines represent the peak-differentiating and imitating line of the spectrum.

X-ray photoelectron spectroscopy (XPS) was utilized to survey the surface composition and chemical state of the as-prepared Ni-Mo-based nanoarrays. Figure 5 shows the XPS spectra of the Ni_2_Mo_1_-nanoarrays. The survey spectrum (Figure 5a) presents Ni 2p, Mo 3p, Mo 3d and O 1s peaks, which confirms the presence of Ni, Mo and O in the Ni-Mo-based nanoarrays. In Figure 5b, there are two major peaks and two satellites peaks, the major peaks at 855.8 and 873.4 eV correspond to Ni 2p_3/2_ and Ni 2p_1/2_. These peaks correspond to Ni^2+^ in 3Ni(OH)_2_·2H_2_O and NiMoO_4_ [33,34]; the small difference between the fitted XPS line and the measured line of the Ni 2p spectrum is due to the formation of Ni(OH)_2_, and the presence of the O 1s peak is due to the formed oxidation and the water molecules that were absorbed to the surface of the nanoarrays [35]. In Figure 5c, the Mo 3d spectrum has two peaks at 231.9 eV and 234.7 eV corresponding to Mo 3d_5/2_ and Mo 3d_3/2_, which is attributed to the Mo^6+^ oxidation state in NiMoO_4_ [36,37]. This is consistent with the results of the XRD.

### 2.2. Electrochemical Characterization of NiMo-Based Nanoarrays

**Figure 6:**
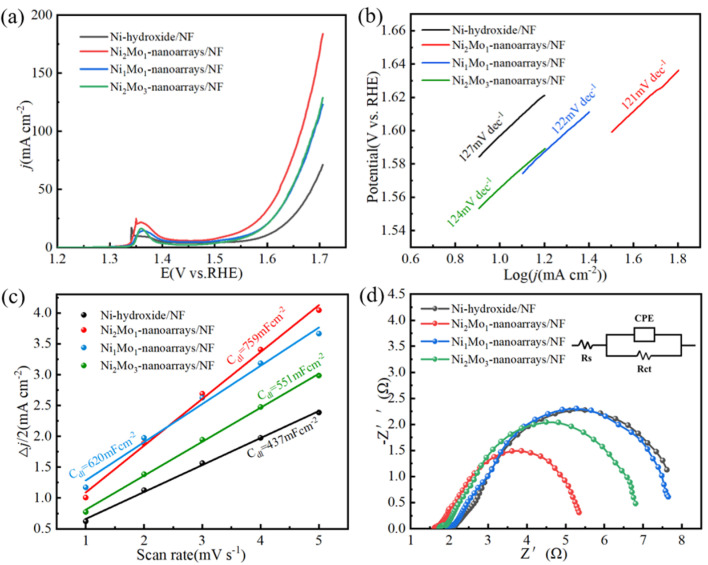
(**a**) LSV plots at 2 mVs^−1^. (**b**) Tafel slopes. (**c**) Double−layer capacitances. (**d**) EIS Nyquist plots. The inset diagram is the equivalent circuit.

The electrocatalytic characterization of NiMo-based nanoarrays for OER was investigated by using them as working electrodes in a standard three-electrode setup in 1M KOH solution. As shown in Figure 6a, Ni_2_Mo_1_-nanoarrays/NF exhibits an overpotential of 296 mV at 10 mAcm^−2^, much superior to Ni-hydroxide/NF (368 mV), Ni_1_Mo_1_-nanoarrays/NF (329 mV) and Ni_2_Mo_3_-nanoarrays/NF (336 mV). This is attributed to the specific surface electronic structure of Ni_2_Mo_1_-nanoarrays/NF that is regulated through the Mo/Ni ratio [30,38]. The peaks at about 1.35 V vs. RHE correspond to the conversion of the Ni(II)/Ni(III) redox process [26,39,40].

Figure 6b shows Tafel slopes of NiMo-based nanoarrays in 1M KOH. As can be seen in the figure, the Tafel slope of Ni_2_Mo_1_-nanoarrays/NF is 121 mV dec^−1^, and it is superior to Ni-hydroxide/NF (127 mV dec^−1^), Ni_1_Mo_1_-nanoarrays/NF (122 mV dec^−1^) and Ni_2_Mo_3_-nanoarrays/NF (124 mV dec^−1^). Ni_2_Mo_1_-nanoarrays/NF shows better OER kinetics than the others. The favorable OER kinetics of Ni_2_Mo_1_-nanoarrays/NF is related to the specific nanoporous network microstructure and coordination of the composition [41].

Double-layer capacitance (C_dl_) is usually used to estimate the size of the active site of catalysts. Figure 6c shows that Ni_2_Mo_1_-nanoarrays/NF has a significantly larger C_dl_ of 759 mF/cm^2^ than that of Ni-hydroxide/NF (437 mF/cm^2^), Ni_1_Mo_1_-nanoarrays/NF (620 mF/cm^2^) and Ni_2_Mo_3_-nanoarrays/NF (551 mF/cm^2^) (Figure 6c). The above results demonstrate that Ni_2_Mo_1_-nanoarrays/NF has more rapid water oxidation and more exposed active sites [20,42]. This is attributed to its unique nanoporous structure.

The interface charge transfer capacity of electrocatalysts can be estimated by EIS [43,44]. According to our results (Figure 6d), Ni_2_Mo_1_-nanoarrays/NF has the smallest semicircle in EIS. The Rct of Ni_2_Mo_1_-nanoarrays/NF (4.18 Ω) is lower than that of Ni-hydroxide/NF (7.52 Ω), Ni_1_Mo_1_-nanoarrays/NF (6.55 Ω) and Ni_2_Mo_3_-nanoarrays/NF (5.71 Ω). This indicates that the electrochemical impedance of Ni_2_Mo_1_-nanoarrays/NF is much lower than the others, and demonstrates the excellent charge transfer capacity of Ni_2_Mo_1_-nanoarrays/NF. This can effectively accelerate the charge transfer between electrolyte interfaces and electrocatalysts [45].

**Figure 7:**
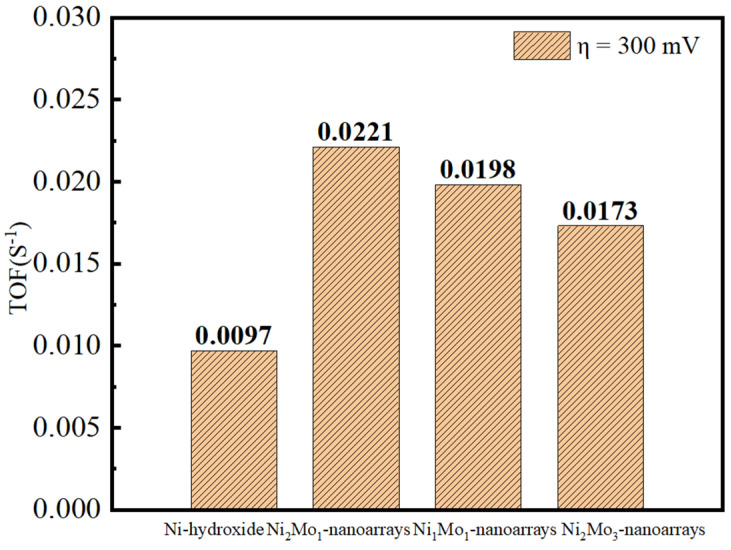
TOF values of NiMo−based nanoarrays under 300 mV overpotential.

The catalytic activity of the electrocatalyst was evaluated by turnover frequency (TOF). The value of the TOF was obtained according to the formula TOF = (AJ)/(4 mF), where A is the active area of the electrode, J is the current density under 300 mV, F is the Faradic constant, and m is the active site number estimated from CV curves [46,47]. The results show that Ni_2_Mo_1_-nanoarrays/NF has a TOF value of 0.0221S^−1^, which is superior to Ni-hydroxide/NF (0.0097S^−1^), Ni_1_Mo_1_-nanoarrays/NF (0.0198S^−1^) and Ni_2_Mo_3_-nanoarrays/NF (0.0173S^−1^) (Figure 7). Thus, the intrinsic catalytic activity of Ni_2_Mo_1_-nanoarrays/NF is best in the specimens, and the better charge transfer property of Ni_2_Mo_1_-nanoarrays/NF may benefit the OER [48].

**Figure 8:**
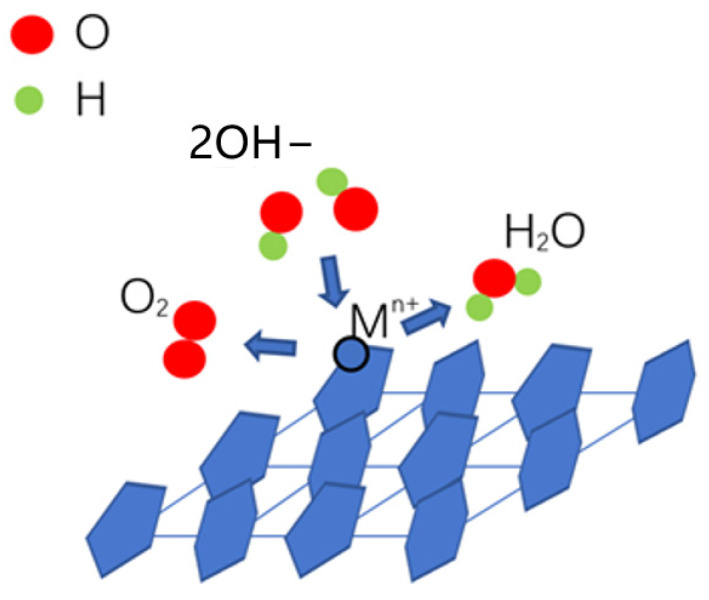
Schematic of the OER mechanism of catalytic activity for metal hydroxides.

During an OER, the surface lattice oxygen atoms of the electrocatalysts are considered to be exchanged with oxygen atoms of the solution. The main metal ions of the electrocatalysts transition from a higher oxidation state to a lower oxidation state and release oxygen [49,50]. The OER in an alkaline electrolyte is considered including several steps [28,51]. In the first step, OH^−^ is adsorbed onto the active sites of the electrocatalyst, and then, [OH] and an electron are released, where [OH] is intermediate. The second step is the combination of [OH] and OH^−^, and [O], H_2_O and an electron are released, where [O] is intermediate. In the third step, [O] and OH^−^ combine to release [OOH] and an electron. In the last step, [OOH] and OH^−^ combine to produce O_2_, H_2_O and an electron [52]. During the OER of metal hydroxides, OH^−^ in the alkaline electrolyte is considered to be adsorbed on the matrix metal ion sites, such as Ni^2+^ on Ni-based hydroxides; there, [OH] is formed [52]. Then, [OH] on the metal sites will react with other OH^−^ in the alkaline electrolyte and release [O], which will combine with other OH^−^ to form [OOH]. Finally, [OOH] will combine with other OH^−^ in the alkaline electrolyte and release O_2_, H_2_O and an electron (Figure 8) [52,53].

Some metal-supported perovskite was prepared as a bifunctional electrocatalyst; the activity of the electrocatalyst was considered to be improved by the bifunctional effect between the oxide-and-metal interface where the electronic property changed [39]. To some NiFe and CoFe catalysts, the formation of O-bridged Fe-M between Fe sites and the nearest-neighbor metal sites seemed to provide stabilized OER intermediates for the high OER activity of MFe oxyhydroxides, while it was unfavorable on pure M-M centers and single Fe sites [40].

**Figure 9:**
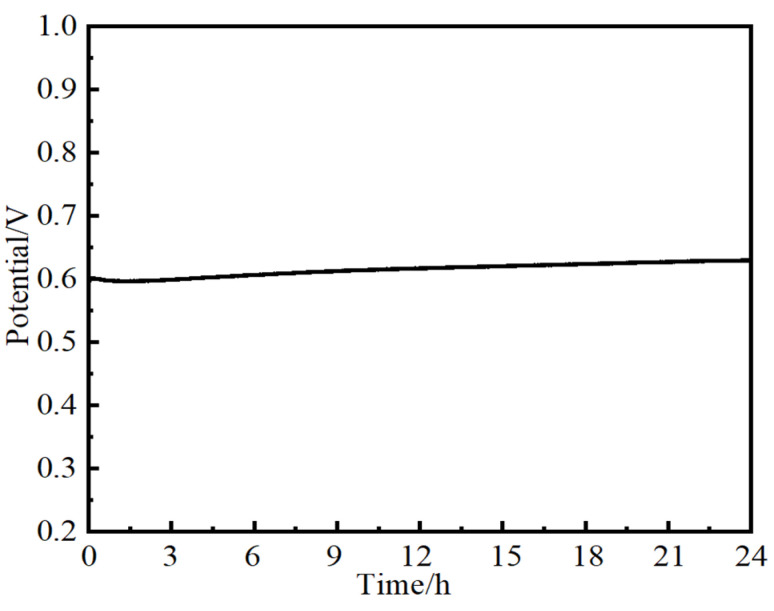
Chronoamperometry test of Ni_2_Mo_1_-nanoarrays/NF at 10 mA·cm^−2^.

**Figure 10:**
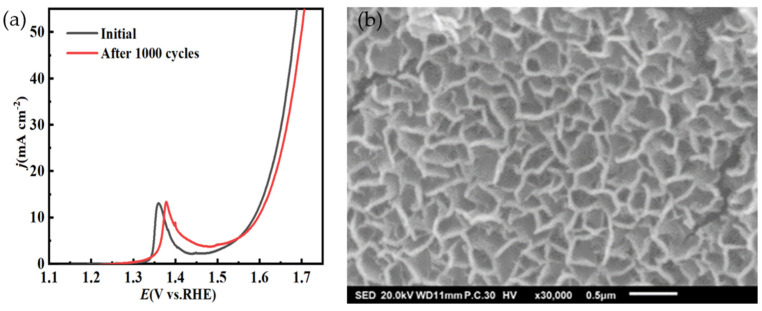
(**a**) LSV curve of initial Ni_2_Mo_1_−nanoarrays/NF and that after 1000 CV cycles. (**b**) Morphology of Ni_2_Mo_1_−nanoarrays/NF after OER test.

To evaluate the potential of NiMo-based electrocatalysts used for practical application, a long-term stability test is necessary. The stability of NiMo-based nanoarray electrocatalysts used for OERs was studied using chronoamperometry tests at 10 mA·cm^−2^ for 24 h in 1M KOH. As shown in Figure 9, a steady V-T curve over 24 h for the Ni_2_Mo_1_-nanoarrays/NF was obtained, authenticating the superior mass transportation and durability of the NiMo-based electrocatalysts [54,55]. The LSV of Ni_2_Mo_1_-nanoarrays/NF presents just a slight amplitude of potential variation after 1000 CV cycles (Figure 10a). After the OER test, Ni_2_Mo_1_-nanoarrays/NF still maintained its inherent morphology (Figure 10b) [56]. These results prove its outstanding durability.

Table 2 shows a comparison of the OER activity of various similar materials. The table shows that the dopants can obviously bring about a morphological change in the electrocatalysts, such as nanoporous, nanoarray and nanosheets in situ on an Ni foam surface. These will introduce an even greater active surface area on the substrate, which can increase the contact area between the electrolyte and nanomaterial. This will help to decrease the overpotentials of the OER.

## 3. Materials and Methods

### 3.1. Materials

Na_2_MoO_4_·2H_2_O (99%), KOH (99%), Ni(NO_3_)_2_·6H_2_O (99%), CO(NH_2_)_2_ and Ni foam (NF, 99.9% purity) were purchased from Aladdin (Beijing, China). Athyl alcohol and HCl were supplied by the Sinopharm Group (Shanghai, China).

### 3.2. Preparation of Ni-Mo-Based Nanoarrays

A piece of Ni foam (NF, 2.5 cm × 2.5 cm) was treated in 1M HCl solution by ultrasonic cleaning for 30 min. After this, the foams were washed by alcohol and distilled water to remove the impurities on the surface. After drying, the foams were used to synthesize a Ni-Mo-based LDH nanoporous structure through a hydrothermal method. Ni(NO_3_)_2_·6H_2_O, Na_2_MoO_4_·2H_2_O and CO(NH_2_)_2_ were dissolved into 50 mL distilled water and continuously stirred for 20 min. The ratio of Ni: Mo ions in the mixed solution varied from 4:1 to 4:2, 4:4 and 4:6. Urea was kept at 15 mmol. The mixed solutions and Ni foams were moved to a 150 mL reactor that had a polytetrafluoroethylene lining and a steel shell outside. Then, they were kept for 4 h at 150 °C. When the reactor cooled down, we took the samples out and washed them with ultrapure water and ethyl alcohol and vacuum-dried them at 80 °C for 10 h.

The OER performance of the electrocatalyst could be regulated by controlling the Ni: Mo ratio. According to the Ni: Mo ratio, the specimens were marked as Ni_4_Mo-nanoarrays, Ni_2_Mo_1_-nanoarrays, Ni_1_Mo_1_-nanoarrays or Ni_2_Mo_3_-nanoarrays.

### 3.3. Structural Characterization

The microstructure and EDS of the electrocatalysts were observed by a field emission scanning electron microscope (JSM7800F, JEOL Ltd., Tokyo, Japan) and a transmission electron microscope (JEM2100, JEOL Ltd., Tokyo, Japan). XRD patterns were detected on a Bruker D8 Advance instrument (Cu Kα radiation, Bruker, Billerica, USA); the scanning speed and the 2θ range were 5°/min and 15–80 degrees. X-ray photoelectron spectroscopy was detected on a Thermo Scientific K-Alpha (Thermo Fisher Scientific, Shanghai, China) instrument.

### 3.4. Electrochemical Performance

The electrochemical experiments were carried out on a Tesco CHI660D(CH Instruments Ins., Shanghai, China) electrochemical work station. The instrument had a standard three-electrode system, which used a platinum electrode as the counter electrode, used an Ag/AgCl electrode as the reference electrode and used the samples as working electrodes. The potentials in this study could be converted into RHEs following the formula ERHE = EAg/AgCl + 0.1989 + 0.0591 × pH. The electrolyte was a 1M KOH solution. The overpotentials were obtained according to the equation η(V) = ERHE−1.23. The LSV curves were measured with a scan rate of 2 mVs^−1^ with 90% iR compensation in the electrochemical work station. Cycle voltammetry (CV) was measured at a scan rate of 1.0–5.0 mV/s. The electrochemical double-layer capacitance (C_dl_) was tested via cycle voltammetry. Electrochemical impedance spectroscopy (EIS) was performed at 1.56 V vs. RHE, and the frequency range was measured from 0.01 to 10^4^ Hz. Stability was investigated by the amperometry technique.

## 4. Conclusions

In summary, the series of nanoporous nickel–molybdenum-based layered double hydroxide electrocatalysts was successfully prepared by a simple hydrothermal process. Benefiting from their nanoporous and nanosheet morphology, the Ni-Mo-based nanoarrays had considerable pore volumes and specific surface areas, which offered more catalytic active sites for the catalytic reaction. The OER performance of the series of Ni-Mo-based nanoarrays was evaluated. The optimal Ni-Mo-based nanoarrays showed a low overpotential of 296 mV for OER at 10 mAcm^−2^, along with a low Tafel slope of 121 mV dec^−1^. The unique nanostructure provided high transfer of electron kinetics in the OER. In addition, the synthesized Ni-Mo-based nanoarrays showed considerable stability in the OER. This work can provide a reference for the design of high-quality and low-cost electrocatalysts.

## Figures and Tables

**Table 1:** Average pore volume and specific surface area of the specimens.

Specimen	Average Pores Volume (cm^3^/g)	Average Specific Surface Area (m^2^/g)
**Ni-hydroxide/NF**	0.016	11.08
**Ni_2_Mo_1_-** **nanoarrays** **/NF**	0.020	14.63
**Ni_1_Mo_1_-** **nanoarrays** **/NF**	0.019	13.30
**Ni_2_Mo_3_-** **nanoarrays** **/NF**	0.019	13.07

**Table 2:** OER activity of this study and other Ni-based electrocatalysts.

Electrocatalyst	Current Density (mA cm^−2^)	Overpotential (mV)	Reference
Ni(OH)_2_	10	595	[41]
NiCo-LDH	10	367	[38]
CoNiAl-LDH	10	303	[43]
NiCo-LDH	10	460	[37]
NiCoSe nanoparticles	10	320	[44]
CoNiB nanorod	10	313	[36]
NiFeW-LDH	10	270	[51]
NiMo-based nanoarrays	10	296	This work

## Data Availability

The data presented in this study are available in the article.
